# Is lactate clearance impaired in septic shock?

**DOI:** 10.1186/s13054-015-1039-x

**Published:** 2015-09-10

**Authors:** Thiago Domingos Corrêa, Jukka Takala, Stephan Matthias Jakob

**Affiliations:** Intensive Care Unit, Hospital Israelita Albert Einstein, Av. Albert Einstein, 627/701, São Paulo, 05652-000 Brazil; Department of Intensive Care Medicine, Inselspital, Bern University Hospital and University of Bern, Freiburgstrasse 10, Bern, 3010 Switzerland

In a recent article in *Critical Care*, Tapia and colleagues evaluated the relationship between exogenous lactate clearance and liver perfusion in a short-term model of endotoxemic shock [1]. The authors concluded that the very low portal-hepatic vein lactate gradient was indicative of the inability of the liver to metabolize the increased lactate load. Although we believe that the authors addressed an important issue, we have some concerns regarding their methodology.

The portal-hepatic vein lactate gradient as a surrogate of liver lactate metabolism may be misleading, since systemic hyperlactatemia increases hepatic arterial lactate delivery and may result in net mesenteric lactate uptake [2]. Given a contribution of 15 % of the hepatic artery to total hepatic blood flow [3], hepatic lactate uptake in endotoxemic animals in the study by Tapia and colleagues (portal vein plus hepatic artery lactate delivery minus hepatic vein lactate efflux) would have roughly doubled during their experiment. At the same time, the net mesenteric lactate uptake (estimated from the product of portal vein flow and portal vein-arterial lactate gradient) increased more than sevenfold. Given the increased mesenteric and hepatic lactate uptake in the study by Tapia and colleagues, we wonder how it is possible that exogenous lactate clearance decreased by a factor of more than 20 during their experiment [1]. Finally, the authors took only six arterial lactate samples until 20 min after completion of L-lactate infusion as opposed to 20 samples with an extension up to 60 min after completion of L-lactate infusion in the original literature [4, 5] and did not present the results, although this was their main finding.
